# Modeling the Friction Behavior of Low-Carbon Steel Sheets Using Various Machine Learning Algorithms Based on Strip Drawing Test Data

**DOI:** 10.3390/ma19061109

**Published:** 2026-03-12

**Authors:** Tomasz Trzepieciński

**Affiliations:** Faculty of Mechanical Engineering and Aeronautics, Rzeszów University of Technology, al. Powstańców Warszawy 8, 35-029 Rzeszów, Poland; tomtrz@prz.edu.pl

**Keywords:** coefficient of friction, friction, sheet metal forming, steel sheets

## Abstract

The application of machine learning (ML) methods enables the modeling of sheet metal friction phenomena based on experimental data, allowing for the prediction of the coefficient of friction (CoF) under various operating conditions. The aim of this article is to compare the predictive capability of a wide range of ML algorithms trained on the results of the strip drawing test. The variable parameters in the strip drawing test were sheet orientation, load, sample orientation relative to the sheet rolling direction, and the drawing quality of the low-carbon steel sheet metal. Based on the coefficient of determination (R^2^) and the root mean squared error (RMSE), it was determined that the best predictive performance was achieved by a trilayer neural network (R^2^ = 0.986, RMSE = 0.0025). It was found that the CoF decreased with increasing countersample surface roughness and load. Meanwhile, the orientation of strip samples relative to the sheet rolling direction had a statistically insignificant effect on the CoF. Based on SHapley Additive exPlanations (SHAP) values, it was shown that the average roughness of the countersamples and the load had the most significant influence on the friction coefficient. This was also confirmed using the F-test and permutation importance analysis of the friction process parameters.

## 1. Introduction

Sheet-metal-forming processes play a key role in the modern manufacturing industry. Conventional sheet-metal-forming techniques [1] as well as incremental sheet-forming methods [2] enable the rapid production of components with complex shapes. The fundamental parameters determining the feasibility of producing stamped parts are the required degree of deformation and sliding friction conditions [3]. Additionally, friction occurring at the tool–sheet interface affects the course of the forming process and the surface quality of the product. Friction is a complex phenomenon dependent on contact pressures [4], the surface condition of the tools and the sheet metal [5], lubrication conditions [6,7], and temperature [8]. This variability significantly complicates both the design of metal-forming processes and their effective optimization. Currently, the following tribological tests are used to investigate friction phenomena in sheet-metal-forming processes: the strip drawing test [6,9], the draw bead test [10], and the bending under tension test [8]. The first of these tests is the most commonly used and at the same time the simplest to perform. It involves drawing a strip of sheet metal between two counterspecimens. The process variables in this friction test most often include unit pressure, temperature, and lubrication conditions [6,9,11].

Classical approaches to describing friction in sheet metal forming processes are most often based on simplified analytical or empirical models that are not capable of fully capturing the nonlinear and multidimensional character of the friction phenomenon [12]. Such models are useful for predicting the value of the coefficient of friction (CoF) under varying formation process conditions. The use of friction models is particularly desirable in numerical simulations based on the finite element method [13,14]. Incorrect or incomplete data lead to discrepancies between simulation results and the actual course of the technological process.

The development of computational methods, including those based on artificial intelligence (AI), opens new possibilities for a more precise description of friction behavior. In recent years, particular attention has been paid to machine learning (ML) methods [15,16], especially artificial neural networks (ANNs) [6,17,18]. Owing to their ability to learn, identify complex nonlinear relationships, and adapt to changing process conditions, these methods constitute a promising tool for analyzing and predicting variations in the CoF [6,17]. The growing popularity of AI-based computational algorithms is reflected in the scientific literature. Szewczyk et al. [19] used the CatBoost machine learning algorithm to predict the coefficient of friction of DC05 steel sheets. The results were consistent with experimental data, with R^2^ values ranging from 0.894 to 0.955. In another study, Szewczyk et al. [6] applied multilayer ANNs with various transfer functions to analyze the CoF of DC04 sheets. The results revealed that the ANN with a log-sigmoid transfer function demonstrated the highest prediction accuracy, with a coefficient of determination of R^2^ = 0.947. Trzepieciński and Szpunar [20] predicted the friction coefficients of Ti-6Al-4V sheets using radial basis function ANNs. The friction model, incorporating contact pressure and lubricant viscosity, achieved a prediction quality of R^2^ = 0.998. Baş and Karabacak [21] employed ANNs, regression trees, and support vector machine (SVM) algorithms to predict the coefficient of friction. The results of performance tests demonstrated that ML-based models successfully predicted CoF variations. Mishra [22] applied several ML regression approaches to predict the CoF of aluminum-based composites. Properly tuned ML models can constitute a powerful tool for predicting CoF in tribological applications. Hasan et al. [23] predicted the CoF of aluminum-based alloys using ML algorithms. A comparative analysis of model performance showed that the k-nearest neighbor (KNN) algorithm exhibited the best predictive capability. Ramesh et al. [24] predicted the CoF of EN AW-6061-based composites using ANNs based on the results of pin-on-disk tests. The errors between the predicted and experimental results ranged from 2% to 5%. Four years later, other authors [25] obtained the same quantitative results and conclusions. Bouchot et al. [26] proposed predicting the CoF of a low-carbon alloy steel using a random forest (RF) algorithm based on the morphological properties of ejected wear particles. It was found that third-body morphology can be successfully implemented for CoF prediction in various tribological situations. The prediction of the coefficient of friction of AlSi10Mg–Al_2_O_3_ composites tested under dry friction conditions was the subject of study [27]. The authors applied the extreme gradient boosting algorithm to predict the coefficient of friction with an accuracy exceeding R^2^ = 0.99. Pan et al. [28] used gradient boosting decision tree and XGBoost algorithms to predict the CoF of high-entropy alloy coatings. A feature engineering strategy was applied to enhance the generalization capability of the ML algorithms. The coefficient of determination of the optimal model reached R^2^ = 0.97. Jogesh [29] proposed an ML framework for predicting the CoF of a nano-lubricant operating under elevated temperatures. The model’s predictions matched the experimental data with R^2^ = 0.92. Volz et al. [30] developed a new method for efficient data collection through time-series analysis of the sliding compression test. Different ML models were trained and implemented in finite element simulations. The authors reported that the use of a feed-forward ANN friction model reduces the error in formation force prediction by approximately 59% compared to simple friction models. The ML approach is an alternative to multiple regression models describing the dependence between loads and the CoF [31]. As demonstrated by Tiruneh et al. [32], different metal-forming processes are characterized by distinct contact conditions, kinematics of the interacting surfaces, and stress–strain states. Therefore, individual approaches should be applied to model friction phenomena; however, the same ML procedures can still be employed.

While machine learning methods have been widely explored in tribology and sheet-metal-formation research, studies focusing specifically on friction modeling based on strip drawing test data remain scarce. Moreover, the available works typically employ a single modelling technique, most commonly artificial neural networks, without systematically evaluating alternative machine learning algorithms. As a result, the relative predictive performance of different ML approaches for estimating the coefficient of friction under sheet-forming conditions remains largely unexplored. This study addresses this gap by performing a comprehensive benchmarking analysis of multiple machine learning algorithms, including several variants of six major ML families (linear regression, regression tree, SVM, Gaussian process regression, ANN, and kernel approximation), for predicting the coefficient of friction based on strip drawing test results. Data-driven prediction of the CoF makes it possible to account for complex relationships among multiple process parameters simultaneously, enabling more precise and flexible friction prediction. Machine learning models can reduce the need for numerous experiments, accelerate process optimization, and provide predictions for new combinations of materials and lubrication conditions, thereby significantly increasing their practical usefulness in the industry. From a scientific perspective, such an approach not only facilitates a better understanding of friction mechanisms in sheet metal formation but also enables the identification of previously non-obvious relationships between material properties, process parameters, and applied lubricants. The results presented in this study have application potential. Accurate prediction of the CoF allows for the optimization of forming technologies, minimization of product defects, and integration with process control systems within the framework of Industry 4.0/5.0 concepts, including the predictive real-time adjustment of production parameters. The manuscript is organized as follows: Section 2 defines the research material and the methodology for the experimental determination of the coefficient of friction, and it also presents the assumptions of the ML approach. Section 3 describes the results and presents a discussion, while Section 4 concludes the paper.

## 2. Materials and Methods

### 2.1. Test Material

Low-carbon steel sheets, due to their high formability, are widely used in sheet-metal-forming processes. For this reason, three different grades of steel sheets were used in the friction tests: drawing quality (DQ), deep drawing quality (DDQ), and extra deep drawing quality (EDDQ). The mechanical properties of the sheets (Table 1) were determined in a uniaxial tensile test at room temperature in accordance with the ISO 6892-1 [33] standard. The tensile tests were carried out along the rolling direction. The surface roughness parameters of the sheets (Table 2) were determined using the Alicona InfiniteFocus (Alicona Imaging GmbH, Gratz, Austria) metrological instrument in accordance with the ISO 25178 [34] standard. Average roughness Sa and root mean square roughness Sq are the primary parameters used to characterize the surface roughness of the sheets. Kurtosis (Sku) and skewness (Ssk) are statistical measures used to describe the shape of the surface height distribution of surfaces subjected to friction.

### 2.2. Friction Testing

**Figure 1:**
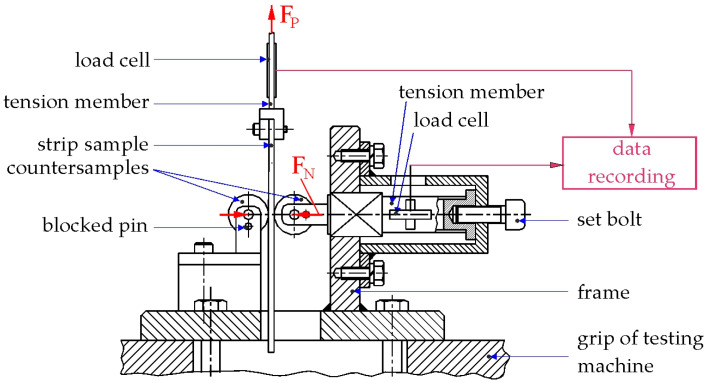
Schematic diagram of the friction tester.

The coefficients of friction of the tested sheets were determined using the strip drawing test. The tribotester used to perform this test consists of a frame with two cylindrical countersamples of 10 mm radius mounted on it (Figure 1). A strip sample with a width of 20 mm is placed between these countersamples. Both countersamples remain stationary during the test to ensure frictional contact conditions.

The frame was fixed in the lower grip of a universal testing machine, while one end of the strip sample was clamped in the upper grip of the testing machine. During the friction test, the clamping force (F_N_) and the pulling force (F_P_) were continuously recorded using load cells (Figure 1) connected to an 8-channel universal amplifier of the HBM QuantumX (Hottinger Baldwin Messtechnik GmbH, Darmstadt, Germany) data acquisition system and a personal computer. The clamping force (F_N_) in the range of 0.25–2 kN was set using an adjusting bolt (Figure 1).

To determine the average CoF, the values of two forces are required, namely the clamping force and the pulling force:

(1) $$\mathrm{CoF}=\;\frac{F_{P}}{{2F}_{N}}$$

The average friction coefficient was represented separately for each experiment. A detailed description of the friction tester design and the procedure for determining the CoF was presented in a previous paper [35].

The surface roughness of the elements forming the friction pair is a key factor determining the value of the CoF [36]. In the present study, three sets of countersamples were planned, with average roughness Ra measured along the generatrix of the samples equal to Ra = 0.32 μm, Ra = 0.63 μm, and Ra = 1.25 μm. The purpose of this approach was to ensure wide data variability for training ML algorithms. Measuring roughness along the generatrix of the samples allows for easy control of countersample manufacturing quality. However, areal surface parameters better reflect the actual condition of the friction pair surface. Therefore, after manufacturing the countersamples, the areal surface roughness parameters were also measured (Table 3).

The steel sheets were manufactured by rolling, which imparted a directional microstructure and surface texture to the sheet surface [37,38]. Therefore, in the experiments, strip samples were cut both longitudinally (L) and transversely (T) with respect to the rolling direction of the sheet metal. To ensure industrially relevant conditions, S100 Plus oil (Naftochem, Krakow, Poland) for deep drawing processes was used. A kinematic viscosity of 360 mm^2^/s at room temperature was determined using an Ostwald viscometer. Prior to testing, the surfaces of both the countersamples and the strip samples were cleaned with acetone and then dried.

### 2.3. Data Analysis Methods

#### 2.3.1. Types of Regression Models

Regression analysis has become one of the foundations of modern data analytics and machine learning. Contemporary approaches to modeling relationships between variables involve complex, nonlinear, and multidimensional interactions that are difficult to capture using classical analytical tools. The dynamic development of methods such as neural networks, decision trees, random forests, and gradient boosting algorithms has enabled the construction of highly flexible models with strong generalization capabilities. These techniques can automatically detect hidden patterns in data, identify significant predictors, and model complex relationships without the need to manually define their analytical form. In this study, a variety of regression algorithms were used to analyze the relationships between friction process parameters (countersample roughness, load, sample orientation relative to the sheet rolling direction, and the drawing quality of the sheet metal) and the value of the CoF. The following regression model types were considered: linear regression (LR), stepwise linear regression (SLR), regression tree (RT), SVM, Gaussian process regression (GPR), ANNs, and a kernel approximation model. For the RT, SVM, GPR, and ANN models, different algorithm structures were examined to better evaluate their approximation capabilities (Table 4). This article does not introduce a new modeling approach but presents a benchmarking study comparing multiple regression algorithms applied to predict the value of the CoF based on the results of the strip drawing test.

**Figure 2:**
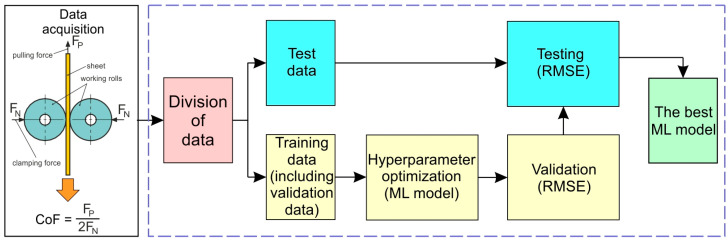
Workflow during data processing.

From the entire training dataset (264 sets), 10% [39,40,41] of the data was randomly selected and assigned to the test set (Figure 2). Although typically 20–30% of the data is allocated to the test set, due to the limited size of the dataset, 10% of the data was randomly assigned to an independent test set, while the remaining data were used for model training and hyperparameter optimization using cross-validation. To evaluate the quality of the models and their generalization ability, i.e., their capacity to predict outcomes for new data, 5-fold cross-validation was applied with the following random data split: 80% for the training set and 20% for the validation set.

In the workflow presented in Figure 2, hyperparameter optimization was performed using the built-in optimization procedure available in Matlab. For each regression algorithm, the model hyperparameters were optimized automatically using Bayesian optimization implemented in Matlab. The optimization aimed to minimize the cross-validated prediction error. The following hyperparameters were optimized depending on the algorithm:

- Linear regression—regularization strength and type of regularization when applicable.
- Regression tree—minimum leaf size, maximum number of splits, and split criterion.
- Support vector machine—box constraint, kernel scale, and the width of the ε-insensitive loss function.
- Gaussian process regression—kernel function parameters such as kernel scale and sigma.
- Artificial neural network—number of hidden neurons and regularization parameters.
- Kernel approximation—kernel scale and regularization parameters.

#### 2.3.2. Characteristics of the Considered Algorithms

LR models are characterized by predictors that are linear in their model parameters, easy to interpret, and that allow for fast prediction. In general, LR models have low predictive accuracy; however, for comparative purposes, this model was included in the analyses. The SLR analysis begins with an initial model and then systematically expands and simplifies it by adding and removing variables based on their impact on the model’s ability to explain data variability.

**Figure 3:**
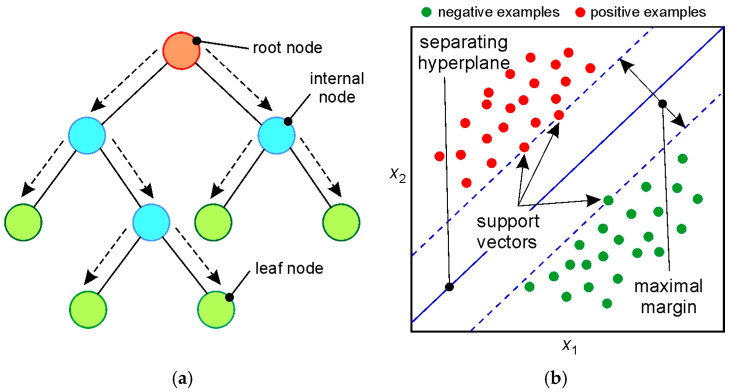
(**a**) Regression tree structure (arrows indicate the processing direction; internal and leaf nodes are marked in blue and green, respectively) and (**b**) classification of data by SVM.

Regression trees are predictive models that estimate the value of a continuous variable by recursively splitting the data into increasingly homogeneous groups. The algorithm selects the feature (predictor) and split threshold that most reduce the prediction error. Each resulting group can be further split until a stopping criterion is met (e.g., a minimum number of observations in a leaf). The example RT shown in Figure 3a predicts the response based on predictors A and B, starting from the top node. Based on the predictor values, the algorithm decides which branch to follow toward a leaf. The response corresponds to the value associated with the leaf. A leaf is a terminal (indivisible) node. Overfitting is controlled by adjusting the “minimum leaf size” parameter. Trees with a small number of observations per leaf are prone to overfitting, whereas a large number of observations per leaf results in lower data fit but reduces the risk of overfitting. For this reason, three types of RT were considered in the analysis: fine tree (minimum leaf size = 4), medium tree (12), and coarse tree (36) (Table 4).

Support vector machines are supervised learning algorithms based on the principle of maximizing the decision margin. In a regression context, the goal is to determine a hyperplane in the feature space that best fits the data or approximates the relationship between variables (Figure 3b) while simultaneously minimizing error and controlling model complexity. The core idea of an SVM is to use only the so-called support vectors—observations that lie closest to the decision boundary and determine its position. By applying a kernel function called “kernel trick”, it is possible to efficiently map data into a higher-dimensional space where a nonlinear problem becomes linearly separable. If *ϕ*(*x*) is the transformation into the higher-dimensional space, the kernel function *K* satisfies the condition:

(2) $$K\left(x_{i},\;x_{j}\right)=\phi (x_{i})\cdot \phi (x_{j})$$

SVMs with different kernel functions, as specified in Table 4, were used to analyze the data. The kernel function determines the nonlinear transformation applied to the data before the SVM is trained.

Nonparametric probabilistic models based on the GPR kernel assume that the underlying function used in modeling can be treated as a Gaussian process. This means that any finite set of function values follows a multivariate Gaussian distribution. The key component of GPR is the kernel function, which encodes assumptions about the function’s smoothness. In this paper, exponential and Matérn 5/2 covariance (kernel) functions were used. The Matérn 5/2 is a kernel that produces moderately smooth predictions. It uses a smoothness parameter ν = 5/2, which controls the smoothness of the resulting function [42]:

(3) $$k\left(r;\nu \right)=\frac{2^{1-\nu }}{\Gamma (\nu )}{\left(\sqrt{2\nu }r\right)}^{\nu }K_{\nu }\left(\sqrt{2\nu }r\right)$$

where *K*_ν_ is the modified Bessel function, *Γ* is the gamma function, and *r* is the distance parameter.

ANNs are a class of machine learning models inspired by the structure of biological nervous systems. Their basic element is the computational neuron, which performs a weighted sum of input signals, adds a bias term, and then transforms the result using a nonlinear activation function. The structure of ANNs consists of an input layer, an output layer, and one or more hidden layers. Connections between layers are defined by weight parameters. Thanks to their ability to approximate any continuous function, ANNs offer high modeling flexibility while maintaining generalization capability. The predictive performance of ANNs strongly depends on the network structure and the number of neurons in the hidden layer(s). Therefore, feed-forward ANNs with different structures (Table 5) were used in this study. The input layer contained four neurons corresponding to countersample roughness, load, sample orientation relative to the sheet rolling direction, and drawing quality of the sheet metal. The output layer consisted of a single neuron representing the CoF.

**Figure 4:**
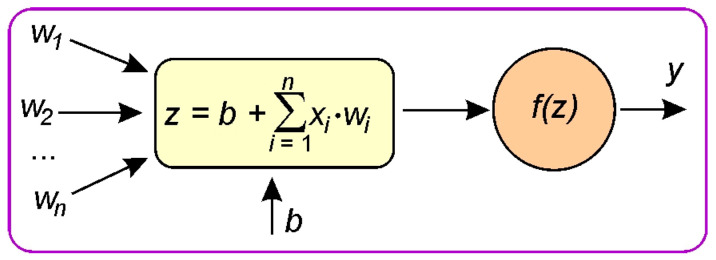
Structure of a neuron in a neural network.

In each neuron (Figure 4), the input *x*_i_ is multiplied by its assigned weight *w*_i_, which indicates the importance of that input. The bias *b* is an additional parameter added to the sum of the weighted inputs, allowing the activation function to be shifted and improving the flexibility of the model. The neuron computes the sum of the weighted inputs and the bias [43]:

(4) $$z=b+\sum_{i=1}^{n}x_{i}\cdot w_{i}$$

The rectified linear unit (ReLU) activation function was used, which is defined as the non-negative part of its argument (Equation (5)). ReLU is one of the most commonly used activation functions for ANNs [44].

(5) $$ReLU\left(x\right)=\max\left(0,\;x\right)=\left\{\begin{array}{c} x\;if\;x>0\\ 0\;if\;\leq 0 \end{array}\right.$$

where *x* is the input to a neuron.

## 3. Results and Discussion

### 3.1. Quality Analysis of ML Models

To evaluate the quality of the ML models, the root mean squared error (RMSE) and the coefficient of determination (R^2^) were used, calculated independently for the validation set and the test set. The RMSE and R^2^ are determined according to Equation (6) and Equation (7) [45], respectively.

(6) $$RMSE=\sqrt{\frac{1}{n}\sum_{i=1}^{n}(y_{p}-y_{t}{)}^{2}}$$

(7) $$R^{2}=\frac{\sum_{i=1}^{n}(y_{t}-\bar{y}{)}^{2}-\sum_{i=1}^{n}(y_{p}-y_{t}{)}^{2}}{\sum_{i=1}^{n}(y_{t}-\bar{y}{)}^{2}}$$

where *y*_t_ is the target value, *y*_p_ is the predicted value, $\bar{y}\;$ is the average value of *y*_t_, and *n* is the number of data points.

Model performance was evaluated using a cross-validation procedure implemented in Matlab (version 2025a). In the k-fold cross-validation (k = 5, default setting), the dataset was partitioned into ten subsets; the model was trained on nine subsets and validated on the remaining subset, and the RMSE was computed from the aggregated cross-validated predictions for all observations.

**Figure 5:**
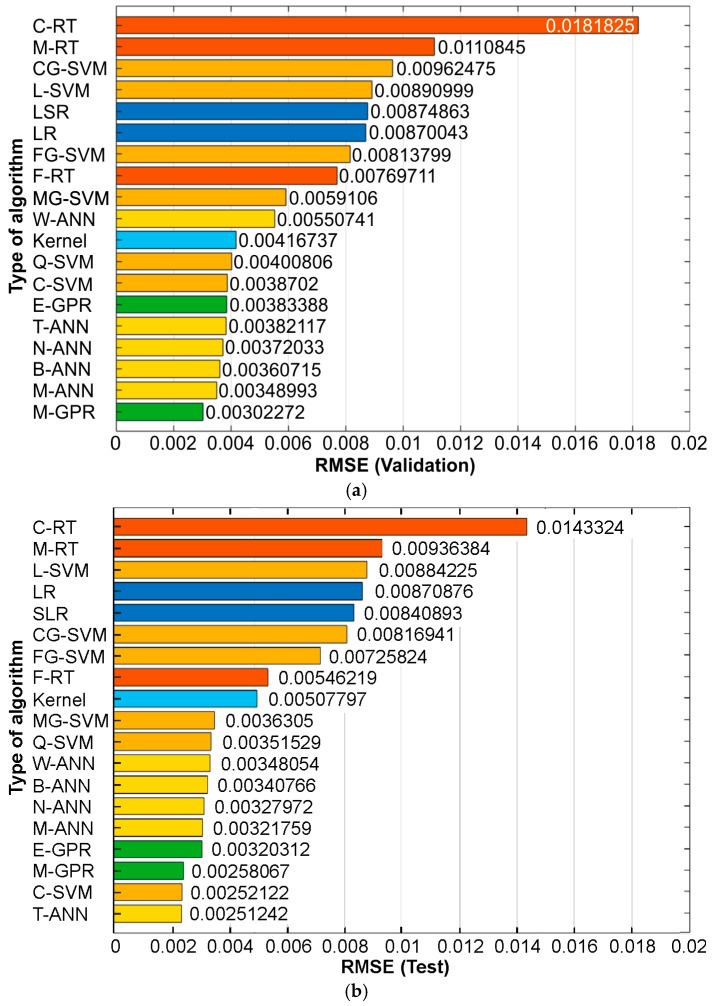
Influence of the algorithm type on the RMSE value for the (**a**) validation and (**b**) test set.

The RMSE is a standard metric used in practical evaluations of regression models, particularly when we want to assess the average prediction error in the same units as the dependent variable [46,47]. The lower the RMSE value, combined with a high R^2^, the better the predictive quality of the model. In general, all regression tree-based models exhibited high RMSE values for both the validation set (Figure 5a) and the test set (Figure 5b). On the other hand, regression models based on Gaussian process regression and ANNs showed relatively low RMSE values. The error values calculated for the test set are more significant for assessing model quality because they were determined using data that were not involved in the training process. The lowest RMSE value, RMSE = 25.1242 × 10^−4^, for the test set was observed for the trilayered neural network (T-ANN) model.

**Figure 6:**
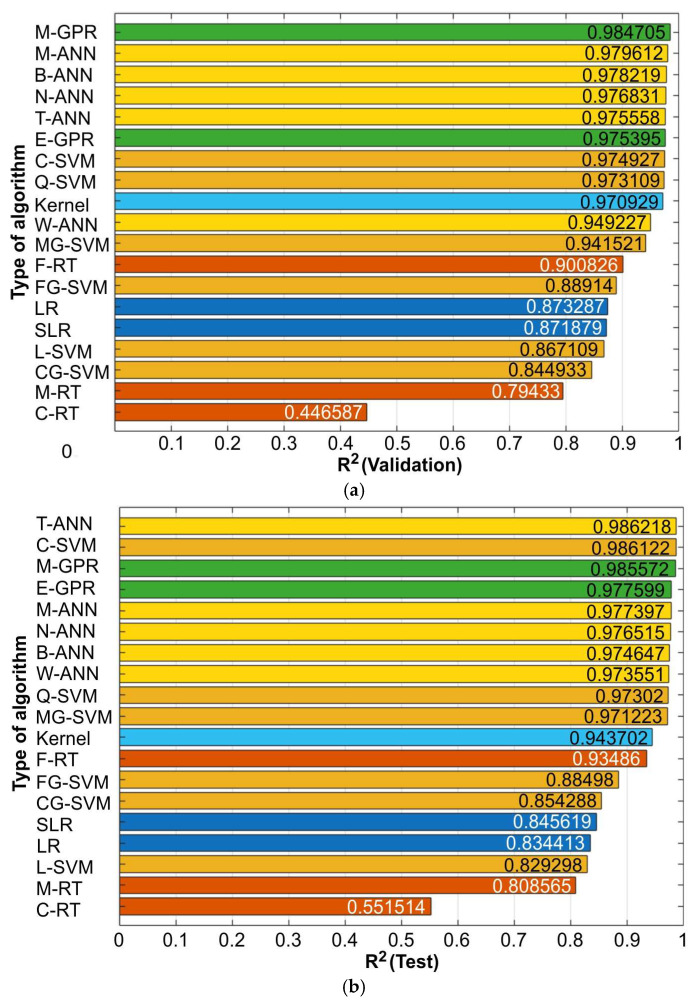
Influence of the type of algorithm on the coefficient of determination R^2^ for the (**a**) validation and (**b**) test set.

Similar to the RMSE values (Figure 5), there is a difference in model quality when assessed based on R^2^ for the validation set and the test set (Figure 6). The higher the R^2^ value, the greater the model’s ability to explain the observed relationships. GPR-based and all ANN-based models demonstrated predictive quality with R^2^ > 0.94 (Figure 6a,b). Among the group of eight models with the best predictive performance, the difference in R^2^ values is 1.177% and 1.284% for the validation set and the test set, respectively. Considering the RMSE (Figure 5) and R^2^ (Figure 6) parameters, the coarse regression tree-based model exhibits significantly lower quality (R^2^ < 0.6, RMSE > 0.014) than the other models. In summary, the best regression capabilities were demonstrated by algorithms based on ANN, GPR, and the coarse support vector machine (SVM) algorithms.

Detailed regression statistics for all analyzed ML models, including the RMSE, R^2^, mean absolute error (MAE) (Equation (8)), mean squared error (MSE) (Equation (9)), and mean absolute percentage error (MAPE) (Equation (10)) parameters, are presented in Appendix A.

(8) $$MAE=\frac{1}{n}\sum_{i=1}^{n}(|y_{p}-y_{t}|)$$

(9) $$MSE=\frac{1}{n}\sum_{i=1}^{n}(y_{p}-y_{t}{)}^{2}$$

(10) $$MAPE=\frac{1}{n}\sum_{i=1}^{n}\left|\frac{y_{p}-\hat{y}}{y_{p}}\right|$$

where $\hat{y}$ is the average value of *y*_p_.

Among all the analyzed algorithms, the best algorithm was selected based on the RMSE and R^2^ values for the test set. The test set is used for an objective assessment of the trained model’s generalization ability, that is, its performance on data that were not used in either the validation or training process. It is employed only at the final stage of modeling to determine quality metrics, such as the prediction error RMSE or the coefficient of determination R^2^, and serves as an independent criterion for model evaluation. The lowest RMSE value (0.00251242) and the highest R^2^ value (0.986218) for the test set were observed for the T-ANN algorithm (Figure 5b and Figure 6b).

### 3.2. Predictive Ability of T-ANN Algorithm

**Figure 7:**
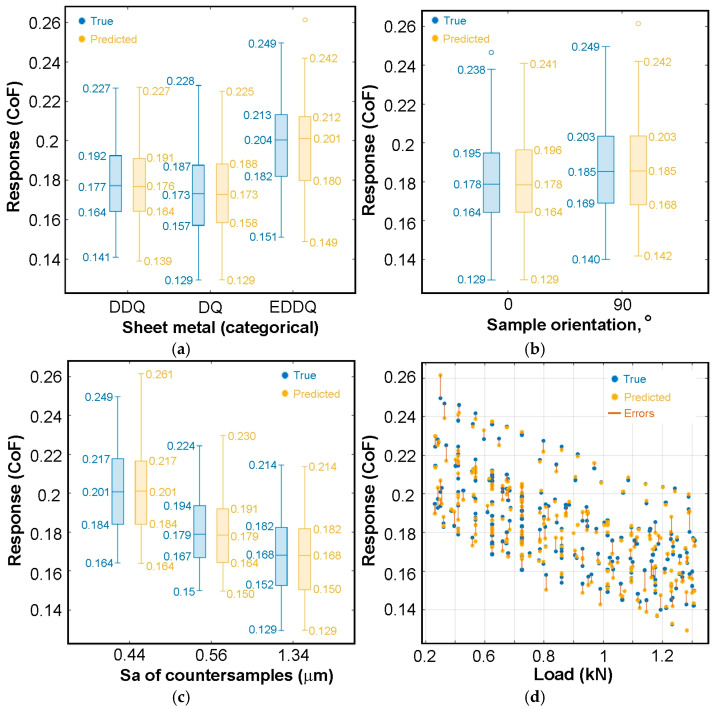
Response box plots for predictions of CoF based on individual input parameters (T-ANN model): (**a**) sheet metal, (**b**) sample orientation, (**c**) Sa of countersamples, and (**d**) load.

The comparison of experimental data with CoF predictions by the T-ANN algorithm based on individual input parameters is presented in the form of box plots in Figure 7. The experimental data regarding sheet metal type (Figure 7a), sample orientation (Figure 7b), and the Sa of countersamples (Figure 7c) exhibit symmetry, meaning the median is located near the midpoint of the box. An increase in the formability of the sheets leads to a higher mean friction coefficient. However, the interquartile range between the first and third quartiles (50% of the data) is similar for all studied sheets (Figure 7a). For the EDDQ sheet, a slightly higher median relative to the mean tolerance value was observed. Samples cut transversely to the rolling direction (90°) showed a higher mean friction coefficient (CoF = 0.186) than strip samples cut along the rolling direction (CoF = 0.179) (Figure 7b). This finding is consistent with previous studies [48,49]. Experimental results also showed that the mean friction coefficient decreases with increasing mean roughness of the countersamples (Figure 7c). A higher profile height limits the area of mechanical interaction between the peaks of the asperities in the friction pair (smaller real metallic contact area). Furthermore, under the lubrication conditions considered in the experiments, a higher profile height provides a larger volume of spaces within the roughness profile, which act as a lubricant reservoir [50]. This leads to more effective lubrication and, consequently, a reduction in the friction coefficient [51,52]. The friction coefficient also decreased with increasing load (Figure 7b), which aligns with results from Al-Samarai et al. [53], Dou and Xia [54], and Severo et al. [55]. Under these conditions, the relationship between the clamping force and pulling force is nonlinear, so the friction coefficient calculated according to Equation (1) decreases with increasing pressure [56]. Regarding the experimental data, only a single measurement was identified as an outlier (Figure 7b).

The maximum error of T-ANN in predicting the friction coefficient values between the first and third quartiles is 0.003 (Figure 7c). Therefore, it can be concluded that T-ANN predicts the position of the middle 50% of the data very accurately. Slightly larger deviations are observed for the whiskers, which represent the data range excluding outliers. While the position of the lower whisker is fairly well predicted, with a maximum error of 0.002 (Figure 7a,b), the maximum prediction error for the upper whisker reaches 0.012 (Figure 7c).

**Figure 8:**
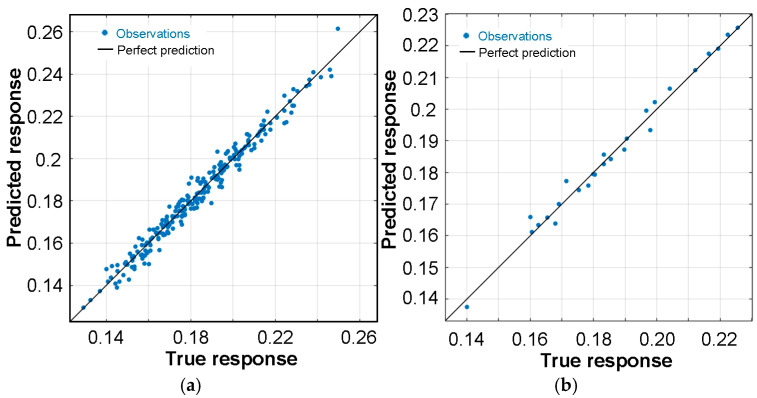
Predicted versus true COF determined for the data contained in the (**a**) training set and (**b**) test set.

As shown in Figure 8, there is a satisfactory correlation between the experimental CoF values and those predicted by the T-ANN algorithm. This applies to data from the validation set (Figure 8a) and the test set (Figure 8b). The data points are distributed proportionally along the perfect prediction line and are clustered around it. This indicates a satisfactory model fit (R^2^ = 0.986 for the test set), as the deviation between the predicted and experimental values was minimal.

**Figure 9:**
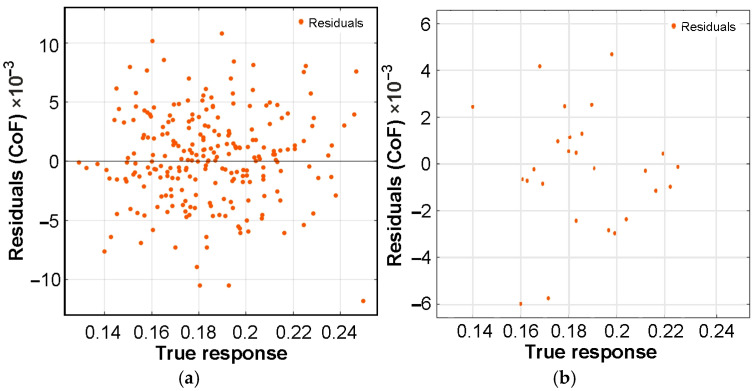
Plots of residuals for (**a**) validation set and (**b**) test set.

The residual distribution plot (Figure 9) allows for the assessment of data homoscedasticity, that is, ε_i_∼N(0, σ^2^), where ε_i_ is the random residual and *σ*^2^ is the variance. The points on the residual plots are scattered randomly around the zero residual value, and a similar random spread is observed in the space defined by the true response.

### 3.3. Feature Importance Analysis

**Figure 10:**
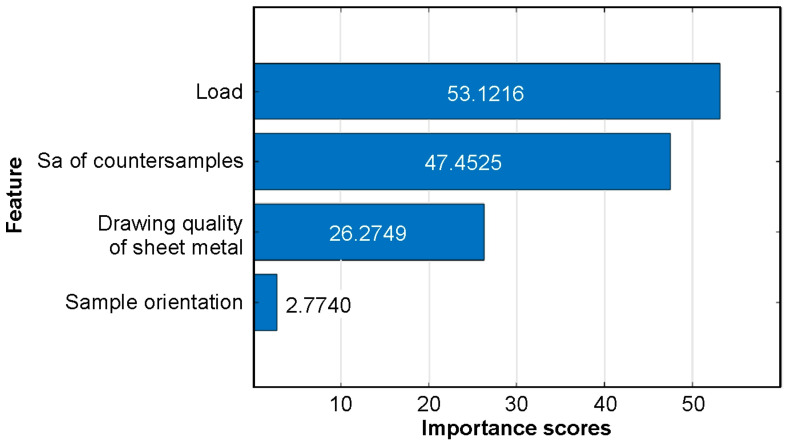
Feature importance scores for T-ANN sorted using F-test algorithm.

The F-test metric assesses how much the model would deteriorate if a specific variable were removed. Each F-test evaluates whether the response values corresponding to different levels of a predictor come from populations with equal means, versus the alternative that at least some of the population means differ. Importance scores correspond to −log(*p*-value). In decreasing order of influence on the CoF, the variables are load, Sa of countersamples, sheet metal grade, and sample orientation (Figure 10).

**Figure 11:**
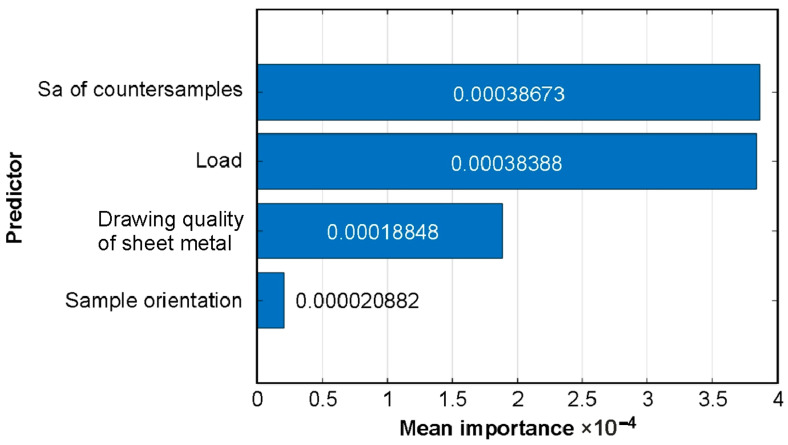
Permutation importance for T-ANN model.

The average influence of predictors in the trained T-ANN model can be assessed based on permutation importance (Figure 11). The mean permutation importance values of predictors allow for evaluating the average impact of predictors on model predictions [57]. The importance of a predictor was assessed by randomly permuting its values and comparing the model’s loss with the original data to the loss after permutation. If a variable carries significant information, its permutation should reduce the model’s performance. In other words, a large increase in model loss with a permuted predictor indicates its importance. The permutation importance of load and the average roughness of countersamples is almost the same (Figure 11). Next in importance are sheet metal grade and sample orientation.

### 3.4. Shapley Additive Explanations Analysis

SHapley Additive exPlanations (SHAP) values are a measure derived from cooperative game theory, used to interpret machine learning models by quantitatively determining the contribution of each variable to a single prediction [58].

(11) $$SHAP\;value=\sum_{S\subseteq N\backslash i}\frac{\left|S\right|!\left(\left|N\right|-\left|S\right|-1\right)!}{\left|N\right|!}\left[f\left(S\cup i\right)-f\left(S\right)\right]$$

where *S* is a subset of features, *N* is the set of all features, *f*(*S* ∪ *i*) is the model prediction for feature *i*, and *f*(*S*) is the model prediction when only the features in subset *S* are present.

**Figure 12:**
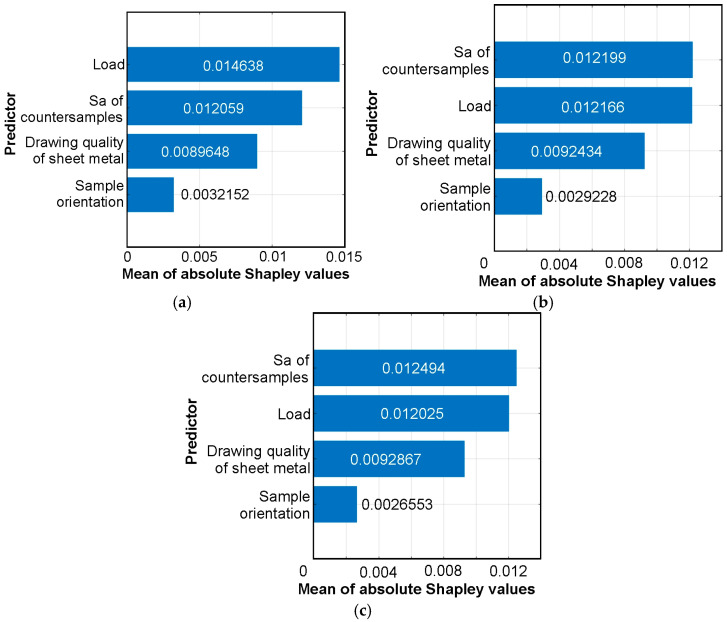
Mean of absolute Shapley values (average impact on the model output magnitude) for (**a**) training set, (**b**) test set, and (**c**) all sets.

For a given observation, the Shapley value represents the average impact of that feature on the model output, calculated as the averaged change in prediction when the feature is added to all possible combinations of the remaining features. Figure 12 shows the mean of absolute Shapley values’ impact on CoF for different datasets. The load variable showed the highest importance (0.014638) in the training dataset (Figure 12a). In the other datasets, the average roughness of countersamples and load carried similar informational importance. It was confirmed that the sample orientation variable had the least impact on the model (Figure 12). The results are consistent with those obtained using the F-test (Figure 10) and permutation importance (Figure 11). For the training set (Figure 12a), the influence of load over the average roughness of countersamples is more dominant than in the test set (Figure 12b). The number of data points in the test set (Figure 12b) was significantly smaller than in the training set (Figure 12a). Thus, for the entire dataset (Figure 12c), the difference in importance between the average roughness of countersamples and load is smaller than when considering only the training set (Figure 12a).

**Figure 13:**
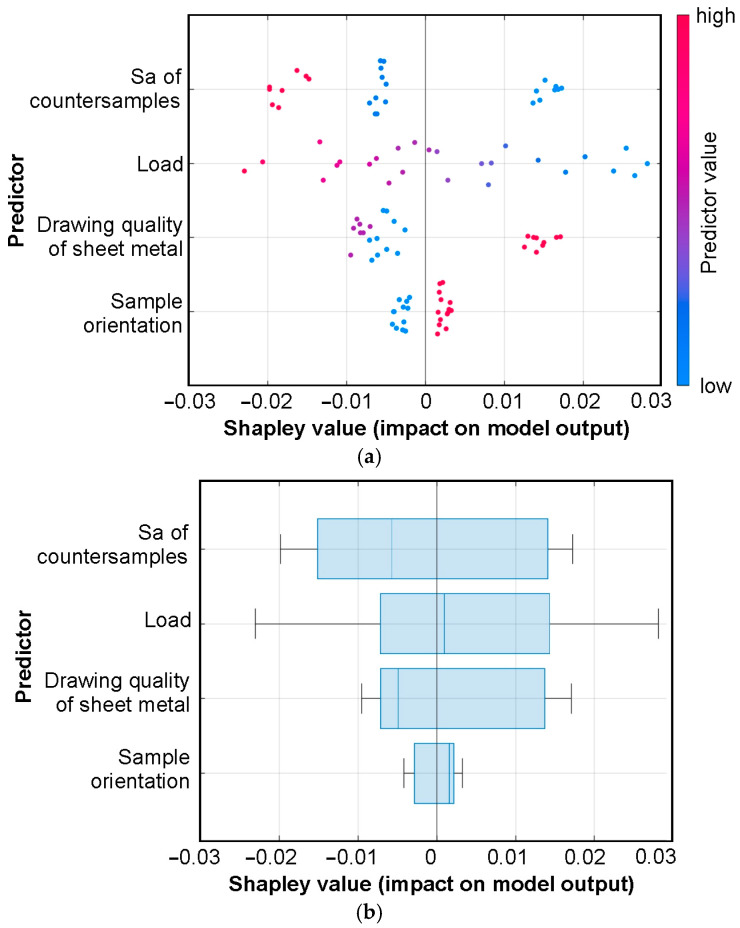
Shapley summary for T-NN model in the form of (**a**) swarm chart and (**b**) box chart.

The dots on the swarm plot of Shapley values (Figure 13a) represent the SHAP value of each observation. The change in SHAP values is indicated using a colormap, where blue represents low values and red represents high values. SHAP values greater than zero indicate a positive influence, while values less than zero indicate a negative influence of the variable on the CoF. Observations are aggregated and represented by point density. The greatest spread is observed for load, which is the parameter that dominates the influence on the friction coefficient. Each Shapley value represents the contribution of a given variable to a single prediction relative to the model’s baseline value.

Low values of the average roughness of countersamples and load have a strong increasing effect on the friction coefficient (Figure 13a). On the other hand, high values of these parameters show little influence on the CoF. The points corresponding to sample orientation are located closest to the axis of the plot, indicating a minimal effect on the CoF. However, samples oriented perpendicular to the rolling direction of the sheet exert a positive influence on the CoF (Figure 13a). Considering the type of sheet metal tested, EDDQ sheets showed the greatest impact on the CoF among all sheet types. The width of the box represents the distribution of Shapley values for a given variable across all observations (Figure 13b). For the variables of load and sample orientation, the median is positive, meaning that on average, these variables increase the CoF prediction (Figure 13b). In the case of the Sa of countersamples, this variable reduces the predicted friction coefficient. The long whiskers corresponding to load indicate a strong influence of this parameter on the CoF, which is also observed from the interpretation of the swarm chart (Figure 13a).

## 4. Conclusions

In this article, an approach to analyzing the results of the strip drawing test using machine learning algorithms is presented. The main results derived from the conducted analyses are as follows:

- GPR-based and ANN-based regression models showed relatively low RMSE values. These models demonstrated high predictive quality, with R^2^ > 0.94. In contrast, regression tree-based models exhibited high RMSE values for both the validation and test sets.
- The RMSE and R^2^ values for the test set were used as the criteria for selecting the best-performing model. The lowest RMSE value (0.00251242) and the highest R^2^ value (0.986218) for the test set were obtained for the T-ANN algorithm.
- A statistical analysis of the experimental and T-ANN-predicted data (box plots) showed that the coefficient of friction decreased with increasing load and average roughness of the countersamples. In general, the data for all predictors exhibited symmetry, meaning that the median was located near the midpoint of the box.
- Feature importance analyses using the F-test, permutation importance, and SHAP values indicated that strip orientation was the predictor least correlated with the CoF. Load and the average roughness of the countersamples showed dominant influence on the CoF.
- The SHAP value swarm plot indicated that high values of the average roughness of the countersamples and load had only a slight effect on increasing the CoF. In contrast, low values of these parameters showed a strong influence on the CoF.

## Figures and Tables

**Table 1:** Thickness and mechanical parameters of the low-carbon steel sheets.

Parameter	DQ	DDQ	EDDQ
Sheet thickness t, mm	1.0	0.8	1.0
Yield strength, MPa	193	196	151
Ultimate tensile strength, MPa	351	336	282
Elongation A, %	0.36	0.42	0.44

**Table 2:** The roughness parameters of the low-carbon steel sheets.

Material	Sq, μm	Sa, μm	Sp, μm	Sv, μm	Sku	Ssk	Sz, μm
DQ	1.67	1.34	5.78	6.75	2.81	−0.11	12.5
DDQ	1.79	1.46	5.81	6.60	2.62	−0.03	12.4
EDDQ	1.83	1.48	6.99	7.27	2.73	−0.11	14.3

**Table 3:** The surface roughness parameters of the countersamples.

Ra, μm	Sq, μm	Sz, μm	Sa, μm	Ssk	Sku
0.32	0.56	69.9	0.44	−0.93	8.43
0.63	0.72	22.04	0.56	−0.27	4.11
1.25	1.57	51.6	1.34	−0.29	3.01

**Table 4:** Regression model types used to model the friction phenomenon.

Algorithm Designation	Algorithm Type	Algorithm Subtype
LR	Linear regression	-
SLR	Stepwise linear regression	-
F-RT	Regression tree	Fine tree
M-RT	Regression tree	Medium tree
C-RT	Regression tree	Coarse tree
L-SVM	SVM	Linear SVM
Q-SVM	SVM	Quadratic SVM
C-SVM	SVM	Cubic SVM
FG-SVM	SVM	Fine Gaussian SVM
MG-SVM	SVM	Medium Gaussian SVM
CG-SVM	SVM	Coarse Gaussian SVM
M-GPR	Gaussian process regression	Matérn 5/2
E-GPR	Gaussian process regression	Exponential GPR
N-ANN	Artificial neural network	Narrow neural network
M-ANN	Artificial neural network	Medium neural network
W-ANN	Artificial neural network	Wide neural network
B-ANN	Artificial neural network	Bilayered neural network
T-ANN	Artificial neural network	Trilayered neural network
Kernel	Kernel approximation	SVM Kernel

**Table 5:** Details of the structure of ANN models.

ANN Designation	Description	Number of Hidden Layers	Number of Neurons in Each Hidden Layer
N-ANN	Narrow neural network	1	10
M-ANN	Medium neural network	1	25
W-ANN	Wide neural network	1	100
B-ANN	Bilayered neural network	2	10, 10
T-ANN	Trilayered neural network	3	10, 10, 10

## Data Availability

The original contributions presented in this study are included in the article. Further inquiries can be directed to the author.

## Appendix A

**Table A1:** Basic regression statistics of the tested models.

Algorithm	RMSE (Validation)	MSE (Validation)	R^2^ (Validation)	MAE (Validation)	MAPE (Validation)	RMSE (Test)	MSE (Test)	R^2^ (Test)	MAE (Test)	MAPE (Test)
LR	0.008700431	7.569750 × 10^−5^	0.87328666	0.007166804	3.82%	0.008708761	7.584252 × 10^−5^	0.83441329	0.007551885	4.05%
SLR	0.008748628	7.653849 × 10^−5^	0.87187888	0.007178003	3.82%	0.008408925	7.071003 × 10^−5^	0.84561904	0.007065668	3.75%
F-RT	0.007697109	5.924549 × 10^−5^	0.90082640	0.006442607	3.53%	0.005462190	2.983552 × 10^−5^	0.93486021	0.004558627	2.46%
M-RT	0.011084474	0.000122865	0.79432996	0.00898849	4.84%	0.009363840	8.768150 × 10^−5^	0.80856529	0.007630125	3.98%
C-RT	0.018182526	0.000330604	0.44658712	0.014809424	8.02%	0.014332369	0.000205416	0.55151424	0.011509386	6.11%
L-SVM	0.008909992	7.938797 × 10^−5^	0.86710902	0.007118325	3.79%	0.008842247	7.818534 × 10^−5^	0.82929822	0.007407036	3.96%
Q-SVM	0.004008064	1.606458 × 10^−5^	0.97310880	0.003064835	1.68%	0.003515289	1.235726 × 10^−5^	0.97302043	0.002792268	1.52%
C-SVM	0.003870204	1.497848 × 10^−5^	0.97492686	0.003003299	1.69%	0.002521223	6.356567 × 10^−6^	0.98612173	0.002026118	1.11%
FG-SVM	0.008137988	6.622685 × 10^−5^	0.88913999	0.005752642	3.17%	0.007258236	5.268199 × 10^−5^	0.88497959	0.004645519	2.61%
MG-SVM	0.005910595	3.493514 × 10^−5^	0.94152054	0.004519262	2.41%	0.003630504	1.318056 × 10^−5^	0.97122292	0.003045925	1.60%
CG-SVM	0.009624752	9.263586 × 10^−5^	0.84493280	0.007446723	3.91%	0.008169408	6.673924 × 10^−5^	0.85428846	0.006641902	3.52%
M-GPR	0.003022715	9.136809 × 10^−6^	0.98470549	0.002388429	1.32%	0.002570669	6.608343 × 10^−6^	0.98557202	0.002157619	1.20%
E-GPR	0.003833880	1.469864 × 10^−5^	0.97539530	0.003041579	1.65%	0.003203116	1.025995 × 10^−5^	0.97759947	0.002586162	1.37%
N-ANN	0.003720328	1.384084 × 10^−5^	0.97683121	0.002905428	1.58%	0.003279718	1.075655 × 10^−5^	0.97651525	0.002546835	1.40%
M-ANN	0.003489929	1.217960 × 10^−5^	0.97961202	0.002709868	1.49%	0.003217591	1.035289 × 10^−5^	0.97739656	0.002463355	1.37%
W-ANN	0.005507414	3.033161 × 10^−5^	0.94922659	0.004129214	2.33%	0.003480542	1.211417 × 10^−5^	0.97355117	0.002793645	1.52%
B-ANN	0.003607152	1.301154 × 10^−5^	0.97821940	0.002770818	1.54%	0.003407656	1.161212 × 10^−5^	0.97464729	0.002783590	1.51%
T-ANN	0.003821166	1.460131 × 10^−5^	0.97555822	0.002951267	1.62%	0.002512421	6.312263 × 10^−6^	0.98621845	0.001874413	1.05%
Kernel	0.004167372	1.736699 × 10^−5^	0.97092863	0.003184646	1.76%	0.005077966	2.578574 × 10^−5^	0.94370208	0.003517190	1.98%
